# Performances of Using Geopolymers Made with Various Stabilizers for Deep Mixing

**DOI:** 10.3390/ma12162542

**Published:** 2019-08-09

**Authors:** Hanifi Canakci, Hamza Güllü, Ali Alhashemy

**Affiliations:** Department of Civil Engineering, University of Gaziantep, Gaziantep 27310, Turkey

**Keywords:** geopolymer, stabilizer, by-product, deep mixing, strength

## Abstract

This research aims to experimentally investigate the potential use of a geopolymer made from various stabilizers or byproducts (fly ash (FA-F, FA-C), slag (SL), glass powder (GP), metakaolin (MK), marble powder (MP), bottom ash (BA), rice husk ash (RHA), silica fume (SF)) to enhance the mechanical performance of soil (clay) via a deep mixing technique. Strengths of geopolymer soilcrete specimens were determined by unconfined compressive strength (UCS) tests regarding curing times (7 to 365 days) by comparing with Portland cement (PC). In addition, ultrasonic pulse velocity (UPV) tests, the effect of molarity (8–16 M), stress-strain behavior, failure modes, and microstructure (SEM, EDX) of geopolymer specimens were examined. Compared to PC, UCS responses of geopolymer specimens yielded: (i) a decreasing trend for FA-F, GP, MK, BA, and MP + FA-F, (ii) an increasing trend for FA-C, SL, and combinations of SL (BA + SL, RHA + SL, SF + SL, MK + SL) favorable with fewer proportions of stabilizers, and (iii) higher increments due to long-term curing (90, 365 days). Despite some decrements, most UCS values were found acceptable (>0.2 MPa) for sufficient enhancement of soft clay. The UCS results were mostly confirmed by UPV performances. The geopolymer specimens were also found to present: (i) strength development for alkaline concentrations from 10 to 14 M, (ii) brittle behavior of stress–strain curves that failed in axial splitting and near axial directions, and (iii) intensity of the silica peak for strength responsibility of the dense microstructure. The findings relatively support the usage of stabilizers or byproducts in the production of geopolymers for potential use in deep mixing. Thus, this research could be a basis for further efforts in this area.

## 1. Introduction

Weak soil such as soft clay, often utilized in construction sites, needs to be enhanced in the mechanism for sufficient bearing capacity, settlement, and permeability of foundations in many civil engineering projects such as dam construction, tunnels, highway embankments, buildings, retaining walls, and so on. [1,2]. Numerous ground improvement techniques have been applied to overcome the difficulties associated with foundations constructed on weak soil. Thus, it is necessary to utilize their advantages for resisting against intolerable limits in foundation design to the maximum extent possible in order to mitigate failure for obtaining economic and useful structures [1,3]. In view of this, deep soil mixing has gained its popularity worldwide since this mixing technique has been effective to increase the strength as well as lessen the compressibility of weak soil. This mixing method essentially results in a soil-binder column by mixing the unstable soil (weak soil) with cementitious and other stabilizers in wet or dry states (i.e., lime, fly ash, bentonite slurry, bottom ash, slag, glass powder, marble dust, silica fume, metakaolin, etc.) [3,4]. By the deep mixing method, it is reported that the improved soil, due to execution of soil mixing columns, could easily meet the adopted design requirements by providing timely, efficient, safe, and cost-effective solutions without challenging excavation and replacement of soil or other expensive methods such as jet grouting and classical drilled piles. This technique also includes several major advantages such as low vibration, low noise, and rapid solidification. Moreover, in a limited workspace, it can be applied over a wide range of soil types including clay, clayey silt, sand, sandy silt, organic clay, and peat soils [3,5]. From surveys of several applications by deep mixing of weak soil (soft soil) summarized in the literature [6], it is observed that Portland cement is the most conventional binder to prove an effective soil-binder column. However, a major defect with Portland cement is its production process that emits a large quantity of carbon dioxide (CO_2_), causing great environmental pollution as well as natural resource depletion. Moreover, the raw materials readily available for cement production are being over-consumed and results in an unsustainable source [7]. In order to reduce environmental impacts, the ground-improvement industry has always been keen on exploring an environmentally friendly, alternative solution with low carbon dioxide emissions for new, viable, and sustainable materials (low-carbon binders) to replace Portland cement as a soil stabilizer. Recently, the use of geopolymers as a green material can be a good option instead of cement for enhancement of weak soil [8,9] by deep mixing [10].

Geopolymers (also known as inorganic polymers) have emerged as a potential alternative material to Portland cement by converting the stabilizers or industrial byproducts of aluminosilicate sources (i.e., slag, fly ash, silica fume, etc.) into a value-added binder using alkaline activators (i.e., mixture of sodium hydroxide (NaOH) or potassium hydroxide (KOH) with sodium silicate (Na_2_SiO_3_) or potassium silicate (K_2_SiO_3_)) formed during the geopolymerization process [11,12,13,14,15]. In this process: (i) amorphous aluminosilicate materials are firstly dissolved by an alkaline activator solution (alkali hydroxide solution and/or alkaline silicate solution) to form reactive silica and alumina [11], then (ii) a polycondensation reaction occurs in a high-alkaline environment by reorganizing aluminum and silica in a more stable Si–O–Al-type structure that provides materials with high mechanical strength and chemical stability [16]. Thus, geopolymers are also able to contribute to applications, in the view of their mechanical properties and durability, using low-cost aluminosilicate materials or even industrial wastes (i.e., fly ash, slag, red mud, bottom ash, and rice husk ash) [17]. Moreover, geopolymers also exhibit excellent adhesions to solid particles and low shrinkage potentials, in which they can be effectively employed in ground enhancement [9,10,18].

Fly ash (using both low calcium and high calcium) based geopolymers are comparable to cement and lime for the improvement (grouting process) of deep soft soils [8,9]. Metakaolin-based geopolymers are found to be an effective soil stabilizer (low shrinkage strain) for clayey soils [11]. Calcium carbide residue as an alkaline activator and fly ash as a precursor can be used to improve the mechanical behavior of silty clay [19] and soft marine clay [20]. Emphasizing the effect of several alkali activators on the strength and microstructure characteristics of soft clay in the grouting process, the strength of soft clay increases because of the slag-based geopolymer [21]. Fly ash based geopolymers often show a slower but longer-lasting strength development (0.3 to 2.8 MPa at 28 days and 5.2 MPa after 90 days) [22]. Stabilization by a slag-based geopolymer (< 8% slag) does not obtain a remarkable strength [23]. The compressive strength of clayey soil stabilized by lightweight alkali-activated slag geopolymer is improved by 200%–350% compared to ordinary Portland cement specimens [24]. Similarly, soil stabilized by slag-based geopolymers indicates a 600% mechanical strength improvement (28 days) [25]. Mix designs and behaviors of metakaolin fly ash based geopolymers are also investigated for comprehensive engineering properties [26]. The mechanical behavior of clay soils is improved using a recycled white glass powder based geopolymer [27]. It is suggested that white glass powder based geopolymers made from recycled calcium carbide residues are useful when high strength and high ductility are required in the soil [28]. From the stability investigations of a geopolymer grout (metakaolin/fly ash binary mixtures), fly ash affects the mechanical properties (at replacement rates of 40% and higher) due to the combined effects of higher packing density and reduced reactivity of the powders [29]. Dependent upon the type of mixture, curing time, and condition of the geopolymer in soil stabilization, it is reported [30] that increasing the binder-to-soil ratio enhances the compressive strength [31]. A higher alkaline activator concentration of the geopolymer reduces the workability and increases the strength [9]. Increasing the molarity of alkali activators and alkali activators/clay improves the compressive strength (0.2 to 4 MPa) of the geopolymer-treated soil (15% binder), which also proposes a good ductility due to the higher energy absorption in all geopolymer specimens [30]. Using fly ash and slag-based geopolymers to improve soft marine clay by deep soil mixing (soil moisture content at 0.75, 1.0, and 1.25 of liquid limit; 10%, 20%, and 30% binders added to the soil by dry soil mass; samples cured for 7 and 28 days) increases the strength (optimum by 20% binder) and proposes a correlation between the strength and modulus of elasticity [10]. Fly ash + silica fume based geopolymers favor flow characteristics for ground modification by grouting [32]. From the review of the literature, it is understood that the geopolymer could be relatively beneficial for ground modification. The literature review indicates that, among many ground improvement methods, using geopolymers especially for grouting and soil stabilization is extensive and increasingly promoted. However, based on the existing gap in the literature and the potential ability of the geopolymer, it is observed that the possibility of using geopolymers for deep soil mixing has had limited attempts in the past [10]. Deep soil mixing treated with geopolymers is a new field with little background and needs further research.

This article aims to experimentally compare the performances of using geopolymers made with various aluminosilicate stabilizers (fly ash (FA, low and high calcium), slag (SL), bottom ash (BA), rice husk ash (RHA), glass powder (GP), silica fume (SF), marble powder (MP), and metakaolin (MK)) for deep mixing of soil (soft clay). Unconfined compressive strength (UCS) and ultrasonic pulse velocity (UPV) (7–365 days) are measured for the specimens of treated soil (i.e., soil-binder (geopolymer) columns). The binary stabilizer combinations are also investigated. To compare the responses of stabilizers, the native cement (Portland cement)-based grout is tested as a control group. Long-term performances (>90 days) of geopolymer-stabilized soils could be worth assessing for the specimens compared to ordinary Portland cement based grout. During UCS testing, the elastic modulus (Es) is also estimated from stress–strain curves, and failure planes of specimens are examined as well. Furthermore, microstructure responses are displayed with the aid of SEM and EDX analyses to elucidate the geopolymer stabilization mechanism. When less liquid content is presented in the geopolymer at the ratio of alkali activator/stabilizer to dissolve the stabilizer, then the mixture collapses instead of flows, and the workability is unmeasurable [31]. Hence, for geopolymer production in deep mixing, the ratio of alkaline activator (Na_2_SiO_3_ + NaOH) to stabilizer for soil-binder specimens is taken in the range from 0.85 to 1.5 on the basis of previous experiences of deep mixing [20,23] and laboratory trials organized in the experimental work of this study.

## 2. Experimental Investigation

### 2.1. Materials

The soil (low-plasticity clay—CL) employed for testing the deep mixing was a fine-grained soil that is classified in accordance with the unified soil classification system (USCS) [33]. It has a liquid limit of 42 and a plastic limit of 24. It is reported that treatment of weak soil via deep mixing is mostly carried out for soils with a water content close to the liquid limit [34,35], which was utilized in this study to prepare all soil-binder specimens. In regard to cement (PC) for the binder of the control group, it was an ordinary Portland cement, type CEM I 42.5R that conformed to specifications [36].

**Figure 1:**
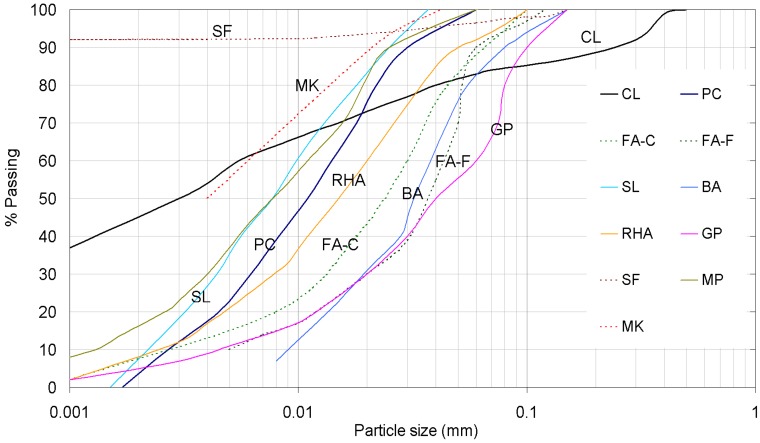
Grain size distribution of materials.

As for the stabilizers in the production of the geopolymer, the fly ash used included both Class F (low calcium content) and Class C (high calcium content) types in accordance with specifications [37]. Class F fly ash possesses pozzolanic properties, while Class C fly ash also has some cementitious properties in addition to pozzolanic abilities [37]. The fly ash Class F (FA-F) is a byproduct of coal combustion collected from Zonguldak Thermal Power Plant (Zonguldak, Turkey). The fly ash Class C (FA-C) is a byproduct from the combustion of lignite or sub-bituminous coals supplied from Soma Thermal Power Plant (Soma, Turkey). All fly ash particles were fine-grained particles ranging from 0.5 to 100 µm in size. The slag (SL) is a byproduct of blast furnace slag (particle size <0.037 mm) that was supplied from an iron industry (İskenderun Iron and Steel Factory, Iskenderun, Turkey). The bottom ash (BA) is a byproduct of coal combustion and was taken from Yatagan Thermal Power Plant (Yatagan, Turkey). The rice husk ash (RHA) is an agricultural byproduct that remains in the combustion process (500 and 600 °C) [38] of rice husks and was taken from a rice factory (Edirne, Turkey). This stabilizer can be considered as green waste with a net zero carbon footprint [39]. High pozzolanic reactions can be obtained when the particle sizes of rice husk ash are smaller (<0.075 mm) [38]. Glass powder (GP) is produced from recycled white glass bottles. To obtain the powder form (specific surface area of 382–388 m^2^/kg), the glass waste was crushed by a crusher and then completely ground by a planetary mill (using a Los Angeles machine). The silica fume (SF) in the experiments is based on silicon dioxide (usually >90%) in the noncrystalline (amorphous) form conformed to the recommended specifications [40]. It has very fine spherical-shaped particles (specific surface area of 21,080 m^2^/kg) with an average diameter size of 0.1 µm (i.e., an ultra-fine powder) that adequately fills the voids between binder particles. The high silica content and ultra-fine powder make silica fumes a highly reactive pozzolanic material for beneficial use in geopolymers [40,41]. The marble powder (MP) (specific surface area 519 m^2^/kg) is obtained as a byproduct from marble stone processing and was from Gaziantep industry. The metakaolin (MK) is obtained from highly processing the kaolinite type of clay (at 700 °C), with most particle grain sizes <0.044 mm. The particle sizes of BA, RHA, GP, and MP used for experiments were below the sieve size of 0.150 mm. Some chemical, physical, and index properties of materials are presented in Table 1. Their particle size distributions are also illustrated in Figure 1.

In regard to the alkaline activators used for obtaining the geopolymer together with the aluminosilicate stabilizers presented above, sodium hydroxide (NaOH) and sodium silicate (Na_2_SiO_3_) were used. Potable water was used to dissolve the pure flakes of NaOH to prepare an aqueous compound with a corresponding concentration of the alkaline solution. The concentration of NaOH employed for all inclusions of soil-binder specimens in this study was mainly 12 M (i.e., 36.09% NaOH + 63.91% water). This has been found to be suitable in laboratory trials and matched with previous studies [42]. Besides, some inclusions were also tested for understanding the effect of concentration (8, 10, 14, and 16 M). The NaOH solution was prepared 24 h prior to mixing. Sodium silicate (Na_2_SiO_3_) was in a liquid gel form that had a chemical composition of 14.7% Na_2_O, 29.4% SiO_2_, and water for the remaining percentage. To add to aluminosilicate stabilizers for geopolymer production, the alkaline solution (combination of sodium hydroxide (NaOH) and sodium silicate (Na_2_SiO_3_)) was prepared in the ratio of (Na_2_SiO_3_/NaOH) = 2.5 (by weight) by blending Na_2_SiO_3_ with NaOH, and then it was left 24 h before use [43,44].

### 2.2. Experimental Pogram

The experimental program of deep mixing in this study is illustrated for Portland cement based grout as a control group in Table 2 and for stabilizer-based geopolymers in Table 3. These mainly included water/binder ratios (0.85–1.25) of PC-based grout, cement replacement (10%–20%), stabilizer proportions (10%–25%), the quantity of binders (PC, geopolymer), quantity of soil and water, and so on.

For performance comparisons of the control group of water-to-binder (w/b) ratios (0.85, 1.05, and 1.25) with the cement replacements (10%, 15%, and 20%) suited to previous studies [44,45] as well as adequacy of geopolymer injection, the ratios of alkaline activator/stabilizer of geopolymer for the specimens of soil-binder columns were similarly taken as 0.85, 1.05, 1.25, and 1.5 with the usable stabilizer proportions 10%, 15%, 20%, and 25%, respectively (by dry weight of soil). The ratio and proportions were based on past suggestions [10,20,23] and laboratory trials. A significant observation should be noted from the laboratory trials: a ratio of alkaline activator/stabilizer less than 0.85 appears to be too difficult for an injectable geopolymer grout for deep mixing. Hence, the efficiency of a high amount of stabilizer proportions (>25%) could become a separate topic to be investigated in future work.

As implied earlier, sample preparations of soft cohesive soils are the most appropriate at water contents close to their liquid limits [34,35]. Other studies also report [21,24] that consistency around the liquid limit provides a homogeneous mixing of soil binder mixture with an alkali solution. Thus, clayey soil for representing in situ weak conditions to be enhanced for deep mixing was mainly prepared with a water content close to the liquid limit (LL) of clay as LL-5 = 37% in the experimental program. Besides, the water contents of LL-10 = 32% and LL-20 = 22% were also tested for some soil-binder specimens to compare the performances of water effects.

### 2.3. Specimen Preparation and Testing Methods

Earlier deep mixing efforts [10,44,45] were followed for sample preparation of soil-binder specimens. For the mixture of the control group (Portland cement based grout), Portland cement was initially mixed with water manually until a uniform grout mixture was achieved. Then, dry soil (clay) was mixed with water (close to the liquid limit) for a few minutes using a rotary mixer machine (having a capacity up to 280 rpm). Next, the grout mixture was added to the soil (clay + water) in the mixer. The grout and soil were mixed thoroughly for approximately 10 min (to avoid any breakage of bonding) at 140 rpm until a homogeneous texture was achieved (soil-binder mixture). A similar procedure was applied for the stabilizer-based geopolymer mixtures, by replacing the geopolymer binder (geopolymer grout = alkaline activator + stabilizer) instead of Portland cement based grout in the soil (clay + water) mixture.

After uniform mixtures (soil + binder mixtures) were achieved, they were cast into a cylindrical plastic mold (diameter of 5 cm, height/diameter ratio of 2.0) in order to obtain soil-binder specimens. All mixtures were compacted in three layers with a manual steel rod in the mold. Manual compaction mainly aimed to eliminate air voids for proper compaction of specimens [21]. The compacted specimens in the molds were immediately wrapped with plastic sheets and maintained in the laboratory control room under ambient conditions (relative humidity of 50%–70%, temperature of 20 °C ± 2) for 48 h. Then, the molds were removed, and the soilcrete (soil-binder) specimens were put in closed plastic bags inside a plastic container in the laboratory room under the same ambient conditions for curing times of 7, 28, 90 and 365 days until testing (i.e., unconfined compressive strength and ultrasonic pulse velocity). The curing times were in accordance with past research [8,46] performed for deep mixing.

Unconfined compressive strength (UCS) tests conducted on soilcrete (soil-binder) specimens (7, 28, 90 and 365 curing days) were performed in accordance with previous specifications [46,47]. A universal compression testing machine (Instron machine with a load capacity of 250 KN; Instron, Norwood, MA, USA) was utilized for the UCS tests under a constant strain rate of 1%/min. The UCS values were measured either in terms of maximum axial stress or stress at 5% axial strain, dependent upon whichever occurred first. From stress–strain curves of UCS tests, the elastic modulus (Es) of soilcrete specimens (7, 28 and 365 curing days) was estimated regarding the tangent modulus conformed in earlier efforts [44]. Ultrasonic pulse velocity (UPV) tests (7, 28, and 365 curing days) were performed for the soilcrete specimens just prior to UCS testing in accordance with specification [48]. All specimens were tested (UCS, UPV) at least three times, and their average values were taken into account for representing their performances. It has been reported [49] that the UCS values can be assessed as a key parameter for measuring the quality of deep mixing. In this research, the UCS performances were considered the main effort of the experimental work to indicate the effect of using geopolymers for treatment of soilcrete specimens by deep mixing. Some UCS values of enhanced soil (0.2–5 MPa for clay, 2–11 MPa for sand) have been proposed by past efforts [4,50], dependent upon binder quantity and soil type. The UPV values would also be beneficial for strength quality of hardened specimens. They can be interpreted by some classifications (very low velocity UPV < 2500 m/s; low velocity 2500–3500 m/s; middle velocity 3500–4000 m/s; high velocity 4000–5000 m/s; and very high velocity UPV > 5000 m/s) given in past work [51]. Finally, the microstructural behaviors of the best UCS responses of hardened soil-binder specimens were investigated via scanning electron microscopy (SEM) and energy-dispersive X-ray spectroscopy (EDX) analyses using a Tescan VEGA 3SB (Tescan, Kohoutovice, Czech Republic) scanning electron microscope operating at 30 KV. After UCS testing, the tested specimens were dried. Small pieces of specimens coated with gold were prepared for SEM and EDX analyses.

## 3. Results and Discussion

### 3.1. Unconfined Compressive Strength (UCS) Performances and Elastic Modulus

**Figure 2:**
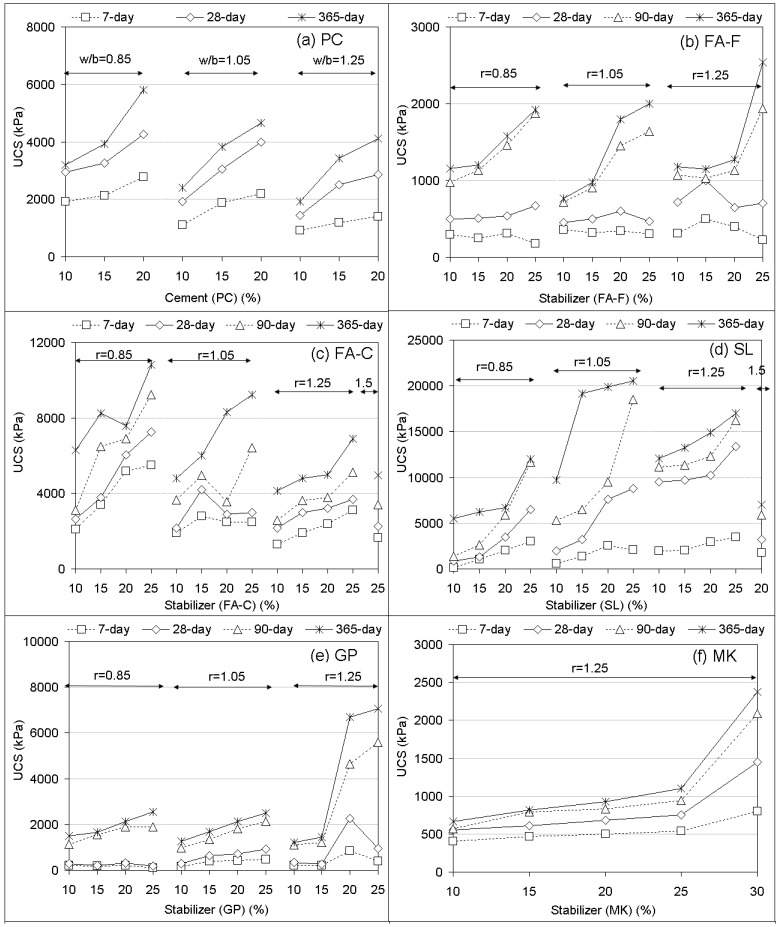

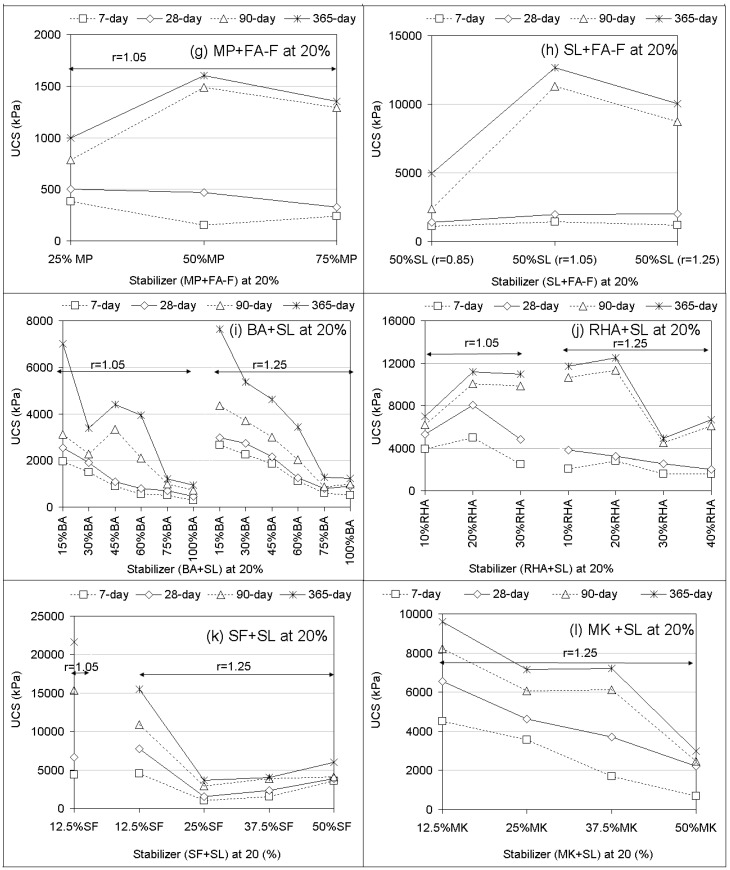
Unconfined compressive strength (UCS) performances of soilcrete specimens for the control group of Portland cement and for the testing group of stabilizer-based geopolymers.

UCS performances of soilcrete specimens regarding curing times are presented in Figure 2. From the control group (PC), it is shown that UCS responses proportionally increased with increasing replacement rates and decreased with increasing w/b ratios. They resulted in the ranges of 915–4270, 1440–5800, and 1914–5912 kPa, respectively, for 7, 28, and 365 days samples. As for the geopolymers compared to Portland cement (PC), it was observed that treatments with stabilizers alone and in combination potentially appeared to produce superior UCS performances (up to 13 MPa for 28-day (SL) and 21.5 MPa for 365-day (SF + SL)) that changed with stabilizer proportions and alkaline ratios (r). On the effect of curing time, both PC and geopolymers resulted in UCSs that increased with increasing curing time. However, it could be emphasized that the majority of stabilizer-based geopolymers were able to develop higher UCS responses specifically for long-term curing (90 and 365 days), likely due to the aging effect of the geopolymer [8] in comparison with the limited increase by PC. It can be said that for most of the stabilizer inclusions alone, UCS responses of geopolymer soilcrete specimens increased with increasing stabilizer proportions and alkaline ratios (r). This was more evident for the UCS performances of long-term curing. In the combinations, it appeared that increasing stabilizer proportions (BA + SL, RHA + SL, SF + SL, and MK + SL) decreased UCSs of geopolymer specimens.

For the geopolymer made with the stabilizers alone, it was found that FA-F produced less UCS performances in comparison with PC. From the results of FA-F, an alkaline ratio of 1.25 appeared more favorable in the production of UCS responses up to 500 (7-day), 1030 (28-day), 1935 (90-day), and 2540 kPa (365-day). The lower strength from use of FA-F could be attributed to its low calcium content (4% CaO) that could be assessed as insufficient for the reorganization of alumina and silica compounds in a more stable Si-O-Al form in order to develop strong bonds at ambient conditions. Specifically, at an early age (7 and 28 days), it could be noted that dissolution of the Si and Al compounds in the alkaline solution was very slow to start the geopolymerization condensation process [52]. Contrary to the FA-F performance, it can be said that the presence of a high amount of calcium may interfere with the polymerization process and alter the microstructure of the specimen [53]. However, even though less UCS responses compared to PC were obtained, it should be emphasized that all UCS magnitudes, due to using FA-F-based geopolymers, could be acceptable to enhance soft soil in accordance with the performance criterion recommended in past work [4,50]. In regard to using the FA-C based geopolymer, the UCS performances were better than the ones from FA-F and PC. They rose to good magnitudes (up to 5520, 7250, 9240, and 10,833 kPa, respectively, for 7, 28, 90, and 365 days at r = 0.85). In most of the FA-C results, UCS values increased with increasing stabilizer proportions and decreased with increasing alkaline ratios. For the strength gain from FA-C, a high calcium (23.9% CaO) content affected the hydration process during geopolymerization reactions (Si-O-Al) and can be considered prominent [53], as emphasized above. Moreover, the contribution of FA-C behavior to soil, such as a mixture of cementitious and pozzolanic materials, could also be significant for the enhancement of soil stiffness [54].

As for the SL-based geopolymer, it was able to achieve relatively higher UCS values than the ones of FA-F, FA-C, and PC, and it even produced superior performances (up to 20,550 kPa for 365 days at r = 1.05). For replacement effects, the UCS responses increased with increasing stabilizer proportions and alkaline ratios up to r = 1.25, but beyond that, they sharply dropped at r = 1.5. Generally, the greater the alkaline ratio (r) the greater the strength from the use of the slag-based geopolymer binder. A relatively rich calcium content (34.2% CaO) of SL as an aluminosilicate precursor in alkali activation, again, can be considered a dominating factor affecting the UCS of the soil-binder (geopolymer) specimen. It is reported [52] that calcium oxide (CaO) of slag produces calcium silicate hydrates (C-S-H) with the aluminosilicate gel enhancing the hardened properties of geopolymerization process. Increasing slag increases the ratio of Si/Al in the geopolymer mixture, which generally leads to an increase in strength. The Si/Al ratio can also influence the effect of the alkaline ratio. Because of the heat of the chemical reaction of calcium in slag, production of sodium aluminate hydrate (N-A-S-H) and calcium aluminate hydrate (C-A-S-H) was accelerated. Then, the silico-aluminate structure was formed in a dense microstructure with an increased strength [55].

In regard to GP, it can be clearly observed that short-term curing performances (7 and 28 days) were low, similar to the ones of FA-F, while long-term curing (90 and 365 days) responses performed better than the ones of FA-F, nearly reaching the good magnitudes of PC. A relatively poor alumina content (1%–1.3%) and low calcium content may be a source for the low performance at an earlier age [56]. Generally, increasing stabilizer rates and alkaline ratios increases strength for long-term performances. From the results of GP, it was clear that the geopolymerization process for obtaining more stable compounds (alumina, silica) mainly occurred during the 90 and 365 days. As a rich source of silica, it can be shown that the GP, due to long-term performances, can be an appropriate substitute for sodium silicate in manufacturing geopolymer grout for soil improvement via deep mixing.

Treatment by MK-based geopolymers for all curing times showed low UCS responses compared to PC. The UCS values remained almost unchanged, with an increasing rate up to the dosage of 25%; beyond that rate, they markedly increased (up to 2375 kPa for 365 days). It appeared that MK offered good enhancement by a large volume of stabilizer replacement and alkaline ratio. However, it should be noted that using MK during specimen preparation showed poor workability because of the relatively high specific surface area and, thus, can be considered a difficult work for deep mixing. Despite the high reactivity due to high alumina (45.7%) and silica (50.6%) contents during alkali activation, the poor workability may lead to low strengths of metakaolin [11]. This may also be a reason for the slightly increased UCS with increasing curing time.

As for the combinations of stabilizer-based geopolymer grouts, it was found that the addition of MP for treatment of the FA-F-based geopolymer in the stabilizer combination MP + FA-F at 20% showed a clear reduction in UCS performance compared to those of FA-F alone. This may be attributed to the fact that a poor reaction developed between MP (natural state) and the alkaline solution due to low alumina and silica contents during geopolymerization. The low strength effects of the nonpozzolanic reaction and noncementation behavior of MP seemed to dominate, similar to some applications in concrete technology [57].

In regard to the addition of SL for enhancement of FA-F-based geopolymer in the stabilizer combination SL + FA-F at 20%, SL was able to yield higher UCS values than the ones of FA-F alone. It was observed that at earlier ages (7 and 28 days), the UCS values of this combination performed well compared to PC. On the other hand, in the long term (90 and 365 days) the addition of SL markedly produced higher responses than PC. The enhancement of FA-F by addition of SL can be explained by enhanced silica-to-alumina and silica-to-calcium ratios in the treated combination [58], as similarly noted earlier. For utilization of FA-F for deep mixing, the combination SL + FA-F at 20% can be offered for practice.

For the ability to use BA alone and together with SL in the stabilizer combination of BA + SL at 20% for geopolymer grout, UCS responses clearly decreased with an increasing proportion of BA. Thus, it was demonstrated that low rates of BA additions for geopolymer grout were favorable for deep mixing applications. The best UCS performances (1950 to 7000 kPa for r = 1.05; 2650 to 7630 kPa for r = 1.25) from 7 to 365 days were obtained by the addition 15% BA (i.e., 15% BA + 85% SL at 20%). Increasing alkaline ratio led to increasing strength. From the results, it can be offered that low rates of BA additions (15%–30%) had similar performances with the ones of PC at early ages of curing (7 and 28 days), while higher responses resulted in the long-term curing period (365 days). Despite having a good pozzolanic activity with high alumina (34.3%) and high silica (39.4%) contents, the decreasing UCS with increasing BA proportions in the geopolymer grout could be explained by some physical effects (very irregular shapes with cavities and pores in fine particle sizes adsorbing water) [59] during the geopolymerization process. Moreover, during the preparation of soil-binder (geopolymer) specimens, it could be emphasized that high amounts of BA additions lead to low workability and low injection ability (high viscosity), similar to the behavior of MK [11], which likely resulted in low strength.

Concerning the utilization of RHA and SL in the combination RHA + SL at 20%, it was observed that RHA was applicable up to 40% addition. Beyond that rate of RHA, the consistency of geopolymer grout became stiff, which is not appropriate for grout injection. UCS performances of 7 and 28 days at alkaline ratio r = 1.25 were comparable with the ones of PC that showed similar responses, whereas most of the remaining RHA additions performed better than PC. This indicated that the RHA (10%–40%) and SL mix is beneficial for geopolymer grout for deep mixing. The best UCS responses in the combination RHA + SL at 20% for both alkaline ratios were obtained by the addition of 20% RHA (up to 11,200 kPa for r = 1.05 and 12,500 kPa for r = 1.25). For enhancement of strength by RHA, it can be said [52] that the inclusion of RHA (limited amount) in geopolymer grout modified the reaction process and physical properties in the geopolymer matrix. The silica content for the geopolymerization process was adjusted by utilization of the silica-rich RHA. The incorporation of RHA with SL increased the reactive silica in the geopolymerization process and formed stronger bonds in the skeleton structure of aluminosilicate compounds. The stronger bonded particles increased the compactness of the microstructure of the geopolymer in a dense state, which increased the hardened properties. In the view of physical influences, it is reported [60] that finer particle sizes of RHA improve reactivity, resulting in a higher degree of geopolymerization. This occurs because the voids formed by free water in the geopolymer structure can be filled by the cellular, porous surface of RHA particles leading to increased strength by the refinement of pore structure and densification of the microstructure of the geopolymer matrix. On the other hand, the increasing amount of RHA (more than 20%) produced low performances due to the increasing SiO_2_/Al_2_O_3_ ratio of the geopolymer matrix, which caused unreactive silica, due to the slower dissolution of Si and Al compounds at ambient temperature, and adsorption of a large quantity of water [52]. Higher additions of RHA were associated with a looser structure of the geopolymer matrix due to the increased amount of unreacted materials [60]. It should be noted that UCS performances of RHA addition at 20% appeared better than the ones of SL alone, specifically for short-term curing, due to contributions to the reactivity of the geopolymerization process as mentioned above.

In regard to the use of SF and SL in the combination SF + SL at 20%, it was observed that 12.5% SF addition gave the best UCS responses (reaching 21,660 kPa for r = 1.05 and 365 days). Beyond that rate (12.5% SF, r = 1.25), the UCS responses sharply dropped, resulting in values that slightly increased with increasing SF, similar to the values of PC. The sharp decrease in UCS response (beyond 12.5% SF) could be attributed to the high surface area of SF adsorbing water during the geopolymerization process, which led to decreased surface friction of soil particles. In addition, a high amount of inactive silica, due to a high amount of SF in the combination SF + SL at 20%, could decrease the reactivity of aluminosilicate compounds because of the slower dissolution [52,61]. Even if the performance was similar to PC, it should be noted that high amounts of SF additions (> 12.5%) led to highly viscous (stiff) grout with less workability that would be difficult to inject. Thus, less SF additions (12.5%) become beneficial for deep mixing applications using geopolymer grout. Silica fumes were able to facilitate the polycondensation reaction. The high performance due to a lower amount of SF addition (12.5%) in combination may be obtained by denser microstructure, which stems from the packing effect of the fine silica fume particles that behave as microfillers by dispersing and filling the inner space inside the microstructure of the geopolymer grout [61]. As a highly reactive pozzolan (92.5% SiO_2_), silica fumes in a lower amount together with SL was able to form a good geopolymerization process that led to a compact microstructure and increased strength [62].

For the addition of MK to SL in the combination MK + SL at 20%, it was found that UCS performances decreased with increasing MK proportions, which mostly produced better performances than PC and MK alone. On the other hand, all UCS responses of the MK addition in combination were less than SL alone, except the one at the earlier age (7 days). As compared with MK alone, it was observed that all MK additions with SL during specimen preparation offered good workability, likely due to particle effects together with the SL combination. Thus, all MK additions in the combination with SL for geopolymer grout can be applied in practice. Compared to PC and MK alone, good UCS performances of MK in the combination may be attributed to the improvement of geopolymer structure because of the good reactivity of MK by the high contents of amorphous alumina and silica compounds. On the other hand, a lower performance, specifically by a high addition of MK in the combination, may be related to the physical effects of specific surface area [63].

From the UCS responses criticized above, it can be emphasized that RHA, MK, and SF have not been employed for treatment of FA-F, since all they have low calcium for strength development during geopolymerization in ambient conditions. They have also not been added to FA-C since they all could potentially cause poor workability and high viscosity for geopolymer grout. As a consequence (Figure 2), most UCS performances of geopolymer grouts can be alternately assessed for deep mixing in place of PC, Whereas SL alone and SL in combination mostly produces favorable UCS responses compared to PC. This is also shown with FA-C. However, dependent upon the stabilizer proportions, fresh characteristics (workability, rheology) of geopolymer grout for its pumping ability should be thoroughly researched and should be a separate topic in future work. The UCS responses of geopolymers via deep mixing (Figure 2) obtained in this study can be compared well with the ones of fly ash in past work [8]. Results of alternate techniques using geopolymers made of metakaolin [11], fly ash, slag [25], glass powder [27,28], and so on, could also be useful for performance assessment. Increasing the stabilizer (fly ash) proportion of the geopolymer specimen improves the strength of stabilized soil [31], similar to FA-C in this study.

**Figure 3:**
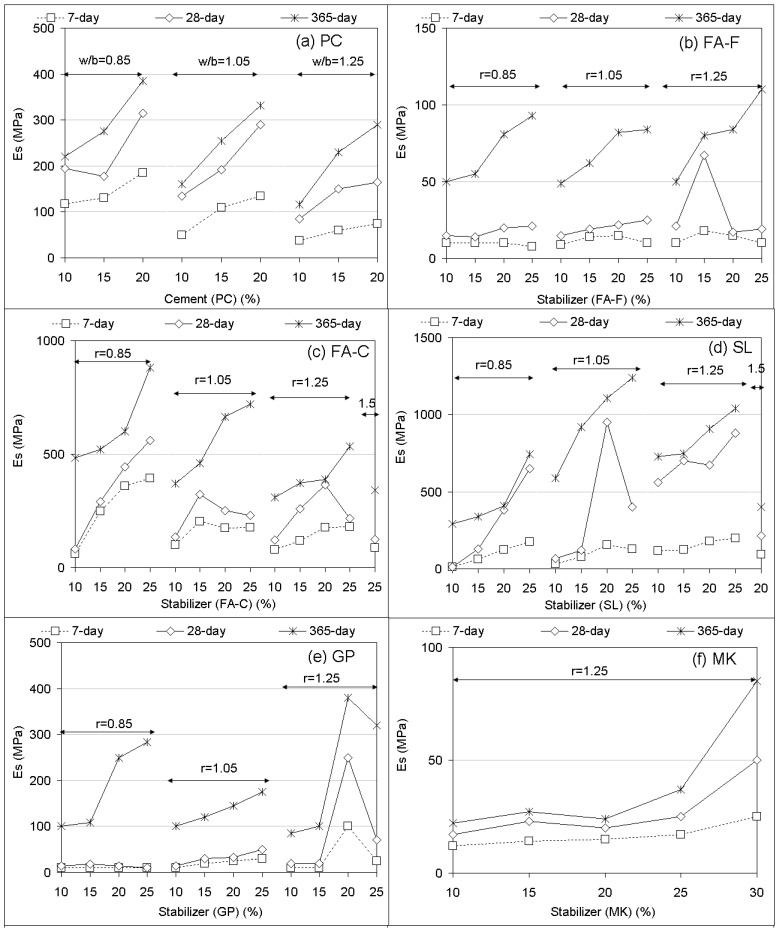

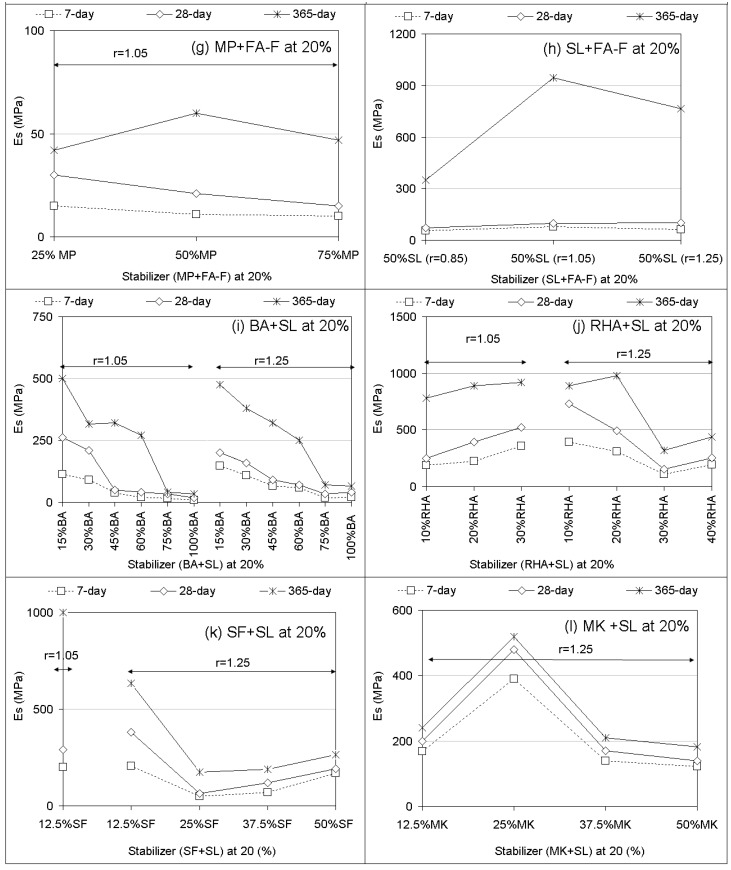
Elastic modulus (Es) performances of soilcrete specimens (PC, geopolymer).

**Figure 4:**
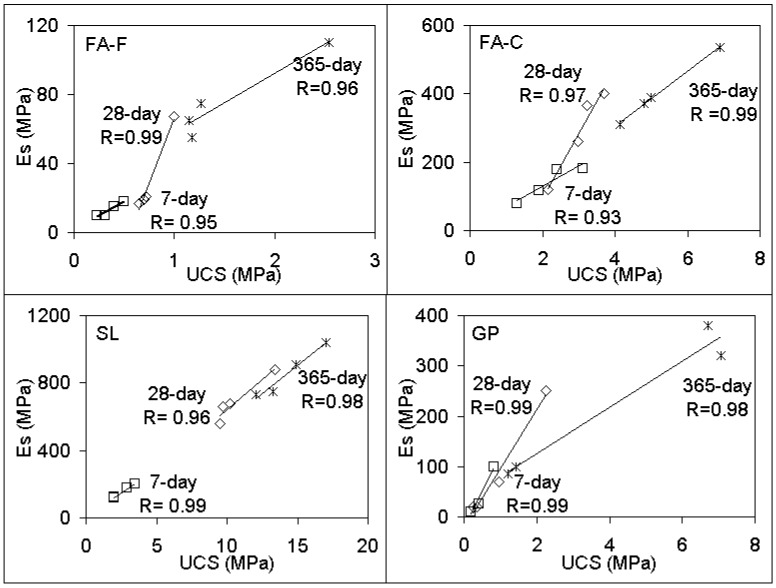
The correlations of Es versus UCS for soilcrete specimens (for FA-F, FA-C, SL, and GP at r = 1.25).

The effects of stabilizer-based geopolymer grout on the elastic modulus (Es) of soilcrete specimens are illustrated in Figure 3. Generally, similar trends to those of the UCS responses (Figure 2) resulted in the elastic modulus. The results were expected since the compressive strength was usually proportional to the elastic modulus. It can be noted that the effect of the Es and UCS of geopolymer grout versus curing time were observed to agree with alkaline and traditional stabilizers researched in previous studies [8,44,64]. For assessing the suitability of responses, the correlations of Es versus UCS values were represented for some inclusions of soilcrete specimens (Figure 4). It was observed that Es increased with increasing UCS, which matched with the general trend of stabilizers proposed in past works [44,65]. As shown from the correlations (Figure 4), the Es correlated mostly well with the UCS values at a relatively strong level (R > 0.9). Stabilizer combinations were also found to potentially offer good correlations. Thus, the fitting curves between Es and UCS could be useful for predicting the strength responses of soilcrete columns by deep mixing in practice in order to provide a better design for experimenters.

### 3.2. Ultrasonic Pulse Velocity (UPV) Performances

**Figure 5:**
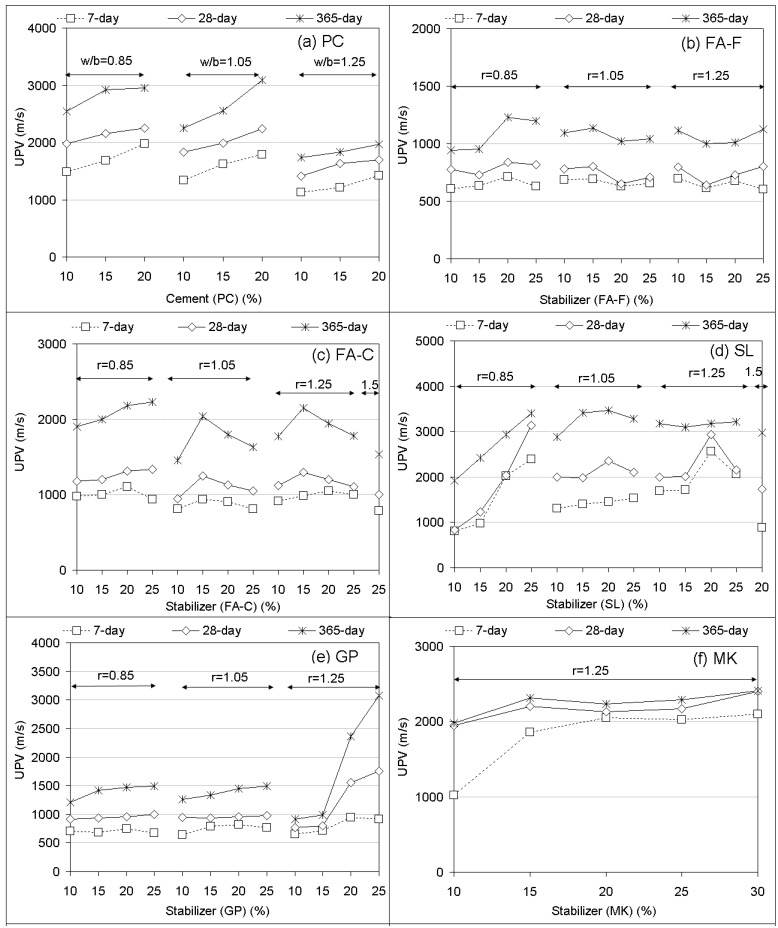

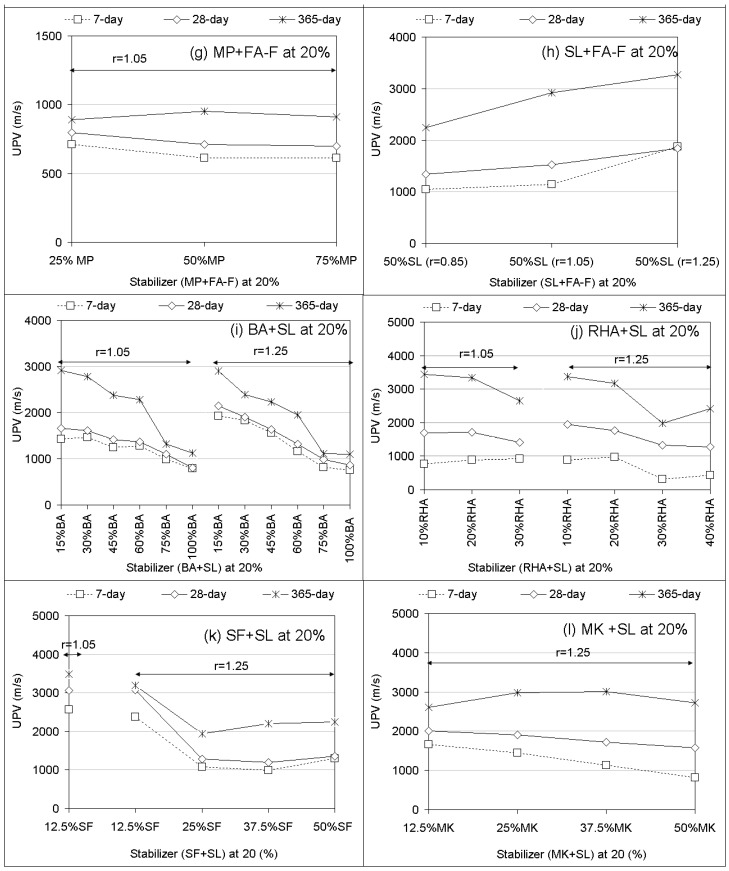
Ultrasonic pulse velocity (UPV) performances of soilcrete specimens (PC, geopolymer).

UPV performances of soilcrete specimens (7, 28, and 365 day) are illustrated in Figure 5. The UPV results can be evaluated in the view of specimen quality regarding the proposed classification [51]. From Figure 5, it is shown that Portland cement produced UPV values in the range 1136–1984, 1415–2450, and 1736–3092 m/s, respectively, for 7, 28, and 365 days specimens. For the stabilizer-based geopolymer grouts, the soilcrete specimens resulted in UPV values in the range 605–2570, 610 –3140, and 890–3488 m/s, respectively, for 7, 28, and 365 days curing times. Based on the results, long-term curing (365 days) appears to improve the quality of soilcrete specimens with geopolymer as well as Portland cement. While few geopolymer specimens can be qualified as “low velocity”, most of them can be assessed in the rank of “very low velocity” for representing the stiffness of specimens. The low to very low magnitude in terms of strength and elastic quality can be mainly explained by the soil type (clay) that aimed to be enhanced in this study. The UPV magnitudes were in agreement with the ones of clay enhancement via deep mixing using grout with added glass powder in a past study [44]. For the effect of curing time, the UPV values seemed to develop slight differences from 7 to 28 d in the majority of stabilizer treatments (FA-F, GP, MK, MP + FA-F, BA + SL, and RHA + SL), while remaining stabilizers notably lead to increasing UPV values with increased curing time. It is clear that the curing of 365-day was more capable of increasing UPV responses.

For Portland cement (PC) replacement, UPV values increased with replacement rate and decreased with w/b ratio (specifically beyond w/b = 1.05). On the effect of stabilizer replacement, FA-F led to geopolymer grouts producing less UPV values, resulting in similar magnitudes with increased stabilizer proportions and ratios of alkaline activator compared to Portland cement. In comparison with FA-F, FA-C was able to yield larger UPV values, generally in an irregular trend, with an increased stabilizer rate and alkaline ratio, but again at magnitudes smaller than PC replacements. Compared to PC and other stabilizers, SL-based geopolymers seemed to result in favorite UPV values that increased with increasing stabilizer replacements up to an alkaline ratio of 1.25. However, beyond that ratio they decreased. Similar to FA-F, geopolymers made with GP mostly produced smaller UPV values, commonly in an irregular trend of increased stabilizer rate and alkaline ratio. MK-based geopolymers with increased stabilizer rates produced better UPV values than FA-F, FA-C, and GP. In addition, they appeared comparable to the ability of PC.

As for the stabilizer combinations, the addition of MP (MP + FA-F at 20%) almost showed no effect on the UPV of using FA-F. On the other hand, the SL addition (SL + FA-F at 20%) with an increased alkaline ratio was able to obtain better UPV values than using FA-F alone. For utilization by the addition of BA (BA + SL at 20%), RHA (RHA + SL at 20%), SF (SF + SL at 20%) and MK (MK + SL at 20%) in combination with SL, it was observed that UPV values mostly decreased with increasing stabilizer proportions and alkaline ratios. They offered favorite UPV values in small replacements (15% BA, 10% RHA, 12.5% SF, and 12.5% MK). As a consequence of UPV results (Figure 5), SF addition at 12.5% (SF + SL at 20%) was found as the most performed UPV response than others. The responses can be followed by the geopolymers made with SL (25% at r = 0.85, 20% at r = 1.25) that seemed to compared well with UCS responses. On the responses of UPV performances (Figure 5), it can be said that when the liquid content of the cured specimen was high, the body wave (shear wave) propagated with a low velocity (i.e., small UPV values) based on wave propagation theory [66]. Moreover, voids of soilcrete specimens, due to particle size and water content, could also lead to poor UPV values that were dependent upon the density of specimens [44]. It was reported [67] that UPV performances decreased with a decreasing density of the specimen.

### 3.3. Effects of Molarity and Water Content

**Figure 6:**
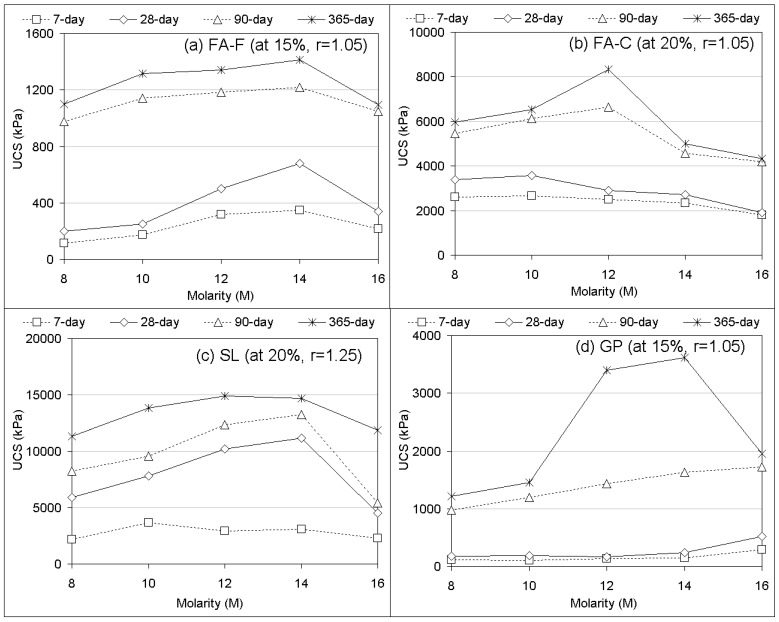
UCS performances of stabilizer-based (FA-F, FA-C, SL, and GP) geopolymer grouts versus molarity.

**Figure 7:**
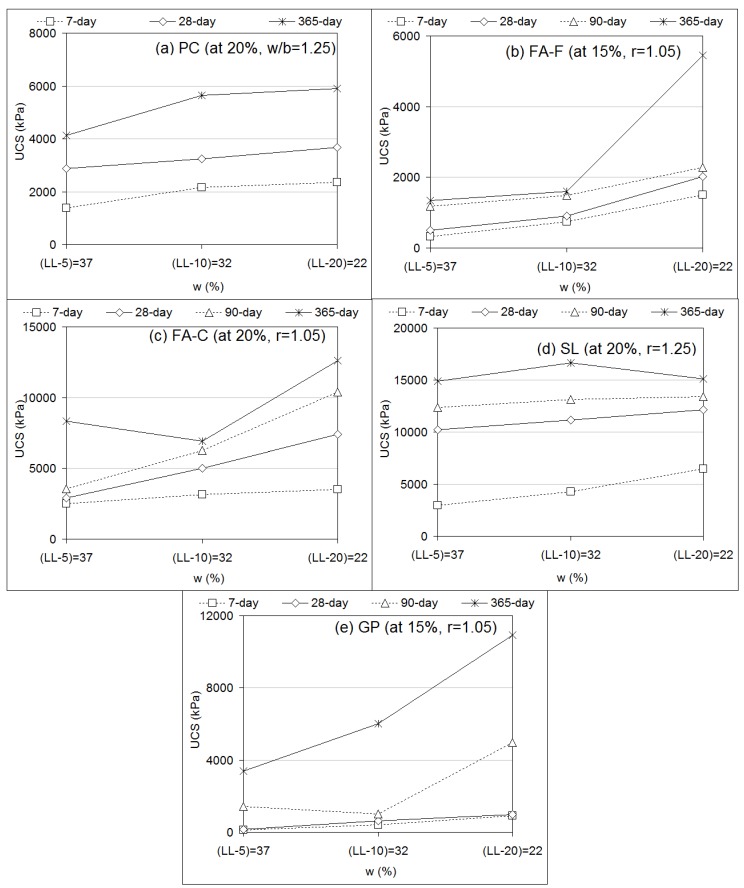
UCS performances of stabilizer-based (FA-F, FA-C, SL, and GP) geopolymer grouts versus water content.

The effects of geopolymer molarity (8 to 16 M) and water content of soil (LL-5, LL-10, and LL-20) in strength performances are illustrated in Figure 6 and Figure 7, respectively. The molarity and water content effects were mainly researched for stabilizers alone (FA-F, FA-C, SL, GP) for some proportions (15%, 20%) and alkaline ratios (r = 1.05 and r = 1.25) randomly chosen for understanding their influences during the geopolymerization processes.

As shown from molarity effects (Figure 6), the stabilizers FA-F and SL tended to increase in strength with an increasing alkaline concentration (molarity) up to 14 M; beyond that concentration the strength obviously decreased. The strength development appeared to be synthesized at NaOH concentrations of 10, 12, and 14 M. Strength of FA-C increased up to 10 (7 and 28 days) and 12 M (90 and 365 days); beyond those concentrations the strength decreased. On the other hand, GP tended to increase with increasing molarity. Generally, it can be seen that the strength performances increased with increasing curing time of the specimens that resulted in a chemical reaction between alumina and silica in the presence of alkali ions [68]. The strength increases with increased alkaline concentration (8–14 M) and may be attributed to the fact that more silica and alumina dissolve in a more concentrated alkaline solution. In the geopolymer reaction, the increasing of concentration will promote an accelerated reaction rate due to the increased concentration of reactants and soluble silicate. The presence of soluble silica improves the condensation process and produces more silica in the polymeric chain that enhances strength properties [60,69]. On the other hand, a concentration of NaOH more than 14 M led to a decline in the strength of the specimen, likely by some negative effects on the geopolymerization process due to: (i) higher viscosity at higher concentrations hindering the leaching of alumina and silica from solid particles [70] and (ii) aluminosilicate gel precipitation at a very early stage coming from the excess hydroxide ion concentration [71]. Specifically, for GP (at 365 days at 16 M), the strength decline could be more dominated by excess Na^+^ ions in the geopolymer matrix [68]. Consequently (Figure 6), alkaline concentration commonly presented strength development between 10 and 14 M; outside this range the strength declined. Even though strength development was reported with increasing alkaline concentration [30], an emphasis should be given that a high alkali activator concentration means a high viscosity, which reduces workability for mixing and the ability of injection for pumping [30,60]. From the experimental work, a general observation was obtained, that the geopolymer specimens activated with the alkaline solutions of 10–12 M represent a favorable alkaline environment for a fluid that also results in acceptable strengths. The concentration effect of NaOH found in this study confirms the influence trends obtained for geopolymers in concrete technology and soil stabilization in previous works [30,68,69].

In regard to the effect of the water content of deep mixing soil (clay) (Figure 7), UCS performances of soil-binder specimens increased with decreasing water content of the soil. The responses were not surprising since water content is the dominant factor that determines the strength of fine-grained material [2]. This is due to the fact [1,2] that quite complex interactions between water and clay minerals lead to the assembly of a soil fabric by frictional forces around soil particles. On the interaction with geopolymer, increasing water content leads to a reduced geopolymerization process (strength development) due to a slower rate of hydration. A higher quantity of water can be immobilized in the geopolymer matrix that causes a significant amount of cation to be absorbed by admixtures [68]. The findings of using various PC and geopolymer grout stabilizers were found to be consistent with the effects of water content researched for different stabilizers for deep mixing of clay in previous studies [44,72].

### 3.4. Stress–strain Curves and Failure Planes

**Figure 8:**
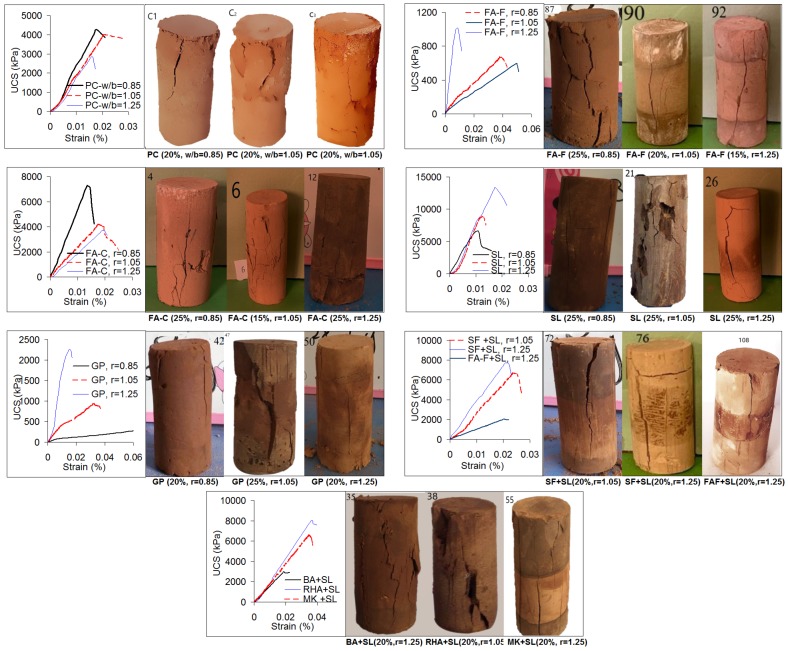
Stress–strain curves and failure planes of soilcrete specimens (for best UCS performances at 28 d).

Stress–strain curves and failure planes of soilcrete specimens are displayed in Figure 8. The failure responses by a description of shear planes are useful for assessing the shear resistance and bearing capacity of soilcrete columns during ground failure. Various failure modes are surveyed for soilcrete specimens such as axial splitting, shearing along a single plane, double shear, multiple fracturing, Y-shaped fracturing, long foliation, and so on [38]. In the studies [38,73] investigating the failure patterns of various cementitious materials and soilcrete specimens under UCS tests, the failure patterns are found to be very close to each other and generally demonstrate failure in axial splitting.

From the results (Figure 8), it was observed that stress–strain curves of all soilcrete specimens (PC, geopolymer grouts) showed a brittle behavior. As for the failure modes, they developed along the shear planes mostly in the axial splitting and near axial orientations, with multiple fracturing, foliations, and localized surface cracks. Failures by axial splitting along foliations were dominant, similar to the soilcrete specimens [38] and cementitious materials [73] previously studied. As a concluding remark, it can be said that the stress behavior and failure modes of soilcrete specimens were not systematically dependent upon the stabilizer inclusions and alkali ratios of geopolymer grout. Following completion of the UCS test, it is worth noting that the soilcrete specimens remained sufficiently stable without fully disintegrating or collapsing. This could be valuable for assessing the damage potential of soilcrete columns in response to mechanism failure. For the sustainable soilcrete specimen without collapse, it can be said [74] that under progressive loading of a compressive test, solid particles were occupied in voids by sliding and shifting, and then they rearranged by leading to denser material towards higher levels of deformation.

### 3.5. Microstructural Evaluation by Scanning Electron Microscopy (SEM) and Energy-Dispersive X-ray Spectroscopy (EDX) Analyses

**Figure 9:**
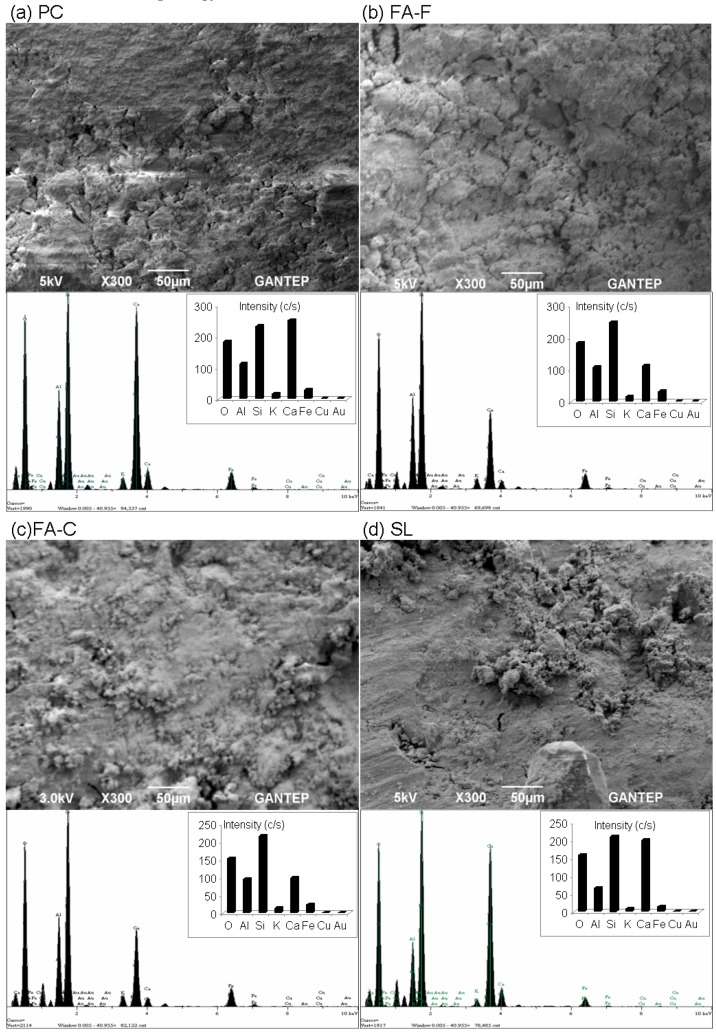

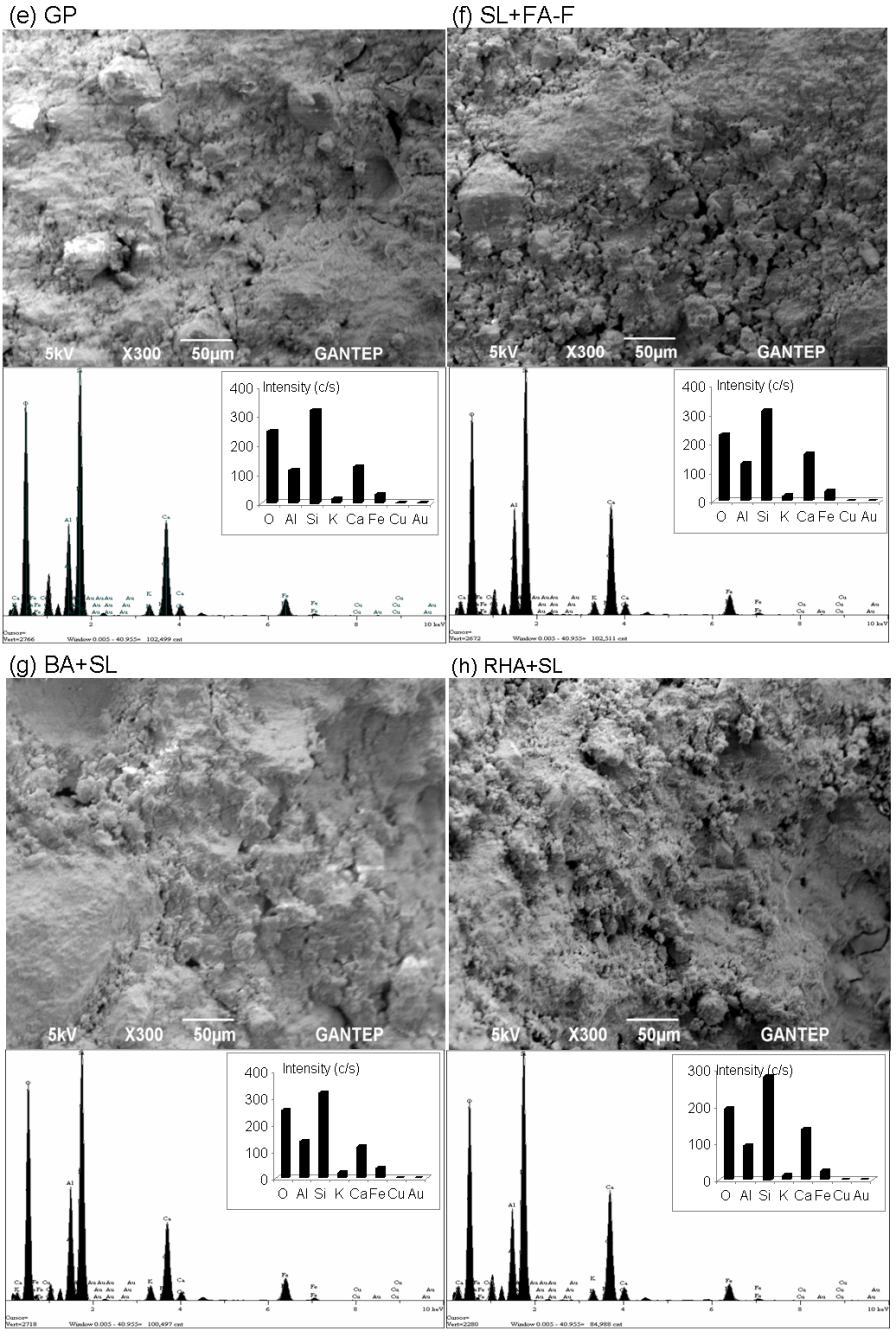

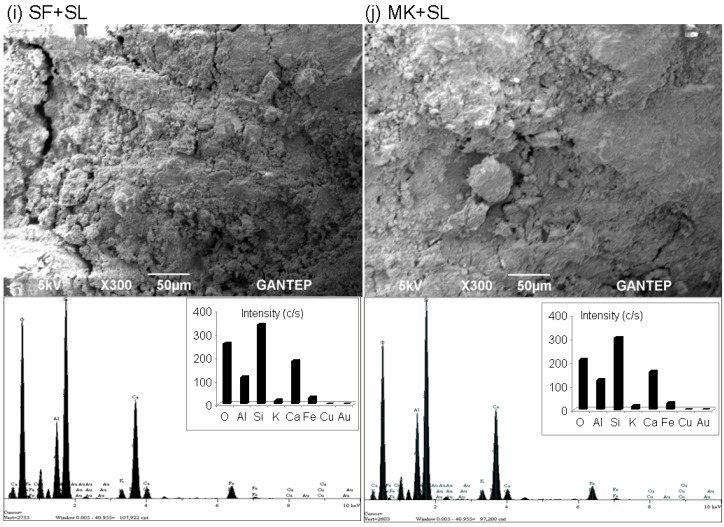
Microstructural (scanning electron microscopy (SEM) and energy-dispersive X-ray spectroscopy (EDX)) analyses of geopolymer soilcrete specimens with stabilizers.

The SEM pictures and EDX patterns of the raw elements of soilcrete specimens are displayed in Figure 9 and regard best UCS performances at 28 days. It can be said that a higher strength geopolymer can be associated with a more desirable internal microstructure [68]. Microstructural analysis (Figure 9) represented a mostly crystalline phase of the geopolymer matrix depending on the chemical and mineral composition of the source materials that could be noticed by the establishment of morphology and reaction. 

Generally, the SEM pictures (Figure 9) visually observed with PC indicate that the stabilizers were stimulated by the alkali activator solution due to internal chemical changes occurring with the particles. Geopolymer stabilizers also comprised particles with irregular shapes in different sizes and rough textures. However, the observation of SEM analysis shows the presence of partially reacted or unreacted solid particles in the geopolymer soilcrete specimens that appear major in FA-F and MK + SL. The larger number of unreacted particles indicates a moderate degree of reaction in the system [75]. Solid particles of soilcrete specimens can be isolated and exhibit large gaps. However, SEM pictures visually compared with PC showed that the microstructure of geopolymer-based stabilizers did not include exaggerate gaps. On the contrary, they tended to have a denser state, probably by rearrangement of particles due to the alkalinity of the binder [30]. However, the unreacted particles during the geopolymerization process, due to the uncompleted chemical reaction of the solid materials in the alkaline environment, could lead to a poorer microstructure with increased porosity [60]. The increased porosity in the geopolymerization system can reduce the strength of the geopolymers, as seen in UCS responses versus geopolymer soilcrete specimens (Figure 2).

As shown from the EDX patterns of soilcrete specimens (Figure 9), the intensity of the calcium peak was dominant by PC-based grout, which is usually expected due to development of a hydration reaction. However, for all geopolymer grouts made with stabilizer alone or combination, the intensity of the silica (silicon) peak likely represents the existence of a pozzolanic effect. This is mostly followed by the peaks of calcium and alumina. The geopolymer specimen of SF + SL in comparison with the remaining inclusions shows the highest intensity of silica peak that offers more favorable UCS responses. Specifically, it is worth emphasizing that the intensity of calcium was highest in the SL-based geopolymer compared to other geopolymer grouts. The elements identified by the EDX test were compatible with the chemical composition of materials (Table 1). The peak with the highest amount of silica within the main compounds may reflect the crystallization of silica in the geopolymer matrix [76]. No alumina and calcium peaks were identified in EDX patterns of geopolymer specimens. This likely suggests that their crystalline phases were only slightly involved in the geopolymerization process and their amorphous phases in the frame of geopolymer matrix [77]. The element peaks decrease in intensity, by EDX patterns, which indicates that the parent materials are not totally dissolved into the inorganic polymeric materials [78]. In general, major crystalline phases occurred in the geopolymer matrix of soilcrete specimens and are responsible for strength. The differences in the crystalline intensities of elements clearly impact the compressive strength of the geopolymer grout. It is reported that the produced strength can be reduced as the intensity of the crystalline elements declines [78]. This EDX pattern seems to agree well with the obtained UCS performances (Figure 2), specifically for the geopolymer grout made with combined stabilizers. However, increased UCS performances due to SL and FA-C resulted from the declined intensity of the elemental compounds that may be attributed to a stronger hydration reaction between the main elements (Ca, Al, Si, and O), similar to PC. Generally, for an increasing UCS of soilcrete specimens, high amounts of Si and sufficient amounts of Ca and Al become appropriate conditions for the formation of the aluminosilicate type of gel and calcium silicate hydrate type of gel in the geopolymer soilcrete specimens by providing a dense structure [27,28].

## 4. Conclusions

This study experimentally uncovers the potential use of geopolymer grouts made with various stabilizers or byproducts (fly ash (FA-F, FA-C), slag (SL), glass powder (GP), marble powder (MP), bottom ash (BA), rice husk ash (RHA), silica fume (SF), and metakaolin (MK)) for ground enhancement of soil (clay) with the use of the deep mixing method. Based on the findings obtained from unconfined compressive strength (UCS) tests, ultrasonic pulse velocity (UPV) tests, stress–strain behaviors with failure planes, and microstructure analyses (SEM and EDX), the following concluding remarks can be proposed for the deep mixing of clay using geopolymer based grouts:

(1) Compared to PC, UCS responses of geopolymer soilcrete specimens were low for FA-F, GP, MK, BA, and MP + FA-F specimens and were high for the specimens FA-C, SL and fewer proportions of SL combinations. Among the geopolymers, SL and SF + SL (12.5% SF) offer the most favorable UCS values, and FA-C follows. The variables affecting the UCS of geopolymer specimens are stabilizer type, proportion, alkaline ratio, and curing time. Es (elastic modulus) values estimated for geopolymer specimens offer strong correlations (R > 0.90) with UCS performances. Thus, they could be beneficial for the stiffness of soil-binder columns during design.
(2) In most of the treatments with stabilizer only, increasing stabilizer proportions (10% to 25%) and alkaline ratios (r = 0.85 to r = 1.25) increases UCS performances with increasing curing time. In the combinations, increasing stabilizer proportions of BA, RHA, SF, and MK added to SL leads to a decreasing UCS.
(3) Increasing curing time proportionally increases UCS for both PC and the geopolymer. However, the effect of long-term curing (90 and 365 days) on UCS of geopolymer specimens results in better performances than PC. In the majority of treatments, due to the geopolymer, UCS performances of long-term curing (365-day) markedly increase compared to those of short-term curing (7 and 28 days).
(4) Most UCS performances of the geopolymer specimens can be sufficiently assessed within the performance criterion of the bearing capacity (0.2–5 MPa) recommended in previous studies. However, the effect of workability, dependent upon the affecting variables, should not be underestimated.
(5) UPV performances mostly confirm the UCS results of geopolymer specimens.
(6) UCS responses of geopolymer specimens increase with alkaline concentrations up to (i) 14 M for FA-F and SL, (ii) 10–12 M for FA-C, and (iii) 16 M for GP. Effects of molarity (8 to 16 M) on the geopolymer specimens (FA-F, FA-C, SL, GP) generally show strength development between 10 and 14 M. UCS increases with decreasing water content of soil (LL-5 = 37 to LL-20 = 22).
(7) Stress–strain curves of geopolymer soilcrete specimens (28 days) show brittle behavior, which develop shear failure planes mostly in the axial splitting and near axial orientations. The behavior and failure modes are not found to be systematically dependent on the stabilizer type, stabilizer proportion, nor alkaline ratio of the geopolymer. Following completion of the UCS test, the geopolymer specimens remain sufficiently stable without fully disintegrating. This could be beneficial to assess the damage potential of soilcrete columns for post-failure responses.
(8) From microstructure analyses (UCS specimens at 28 days), the intensity of the silica peak is identified in all geopolymer specimens. No alumina and calcium peaks are found. The peak of silica appears to be in good agreement with the strength development, specifically for the geopolymer specimens with combined stabilizers (SL + FA-F, BA + SL, RHA + SL, SF + SL, and MK + SL).

## Figures and Tables

**Table 1:** Chemical, physical, and index properties of materials used in the study.

**(a) Chemical composition**											
**Constituent (%)**	**CL**	**PC**	**FA-F**	**FA-C**	**SL**	**BA**	**RHA**	**GP**	**SF**	**MP**	**MK**
**CaO**	18.24	62.58	4.24	23.9	34.2	6.13	1.14	8.21	1.35	52.45	0.2
**Al_2_O_3_**	6.36	5.31	24.4	7.97	10.6	34.3	0.54	1.00	0.39	0.39	45.7
**Fe_2_O_3_**	10.7	4.04	7.1	5.34	1.28	15	0.16	0.52	1.21	0.78	0.31
**SO_3_**	0.08	2.73	0.29	3.03	0.68	0.9	0.25	0.06	1.0	0.076	0.14
**MgO**	0.44	2.82	2.4	0.53	7.63	1.57	0.5	0.14	2.23	0.54	0.23
**SiO_2_**	17.2	20.25	57.2	18.27	40.4	39.4	87.2	78	92.5	1.29	50.6
**K_2_O**	1.49	0.92	3.37	1.39	-	1.19	1.94	0.09	0.08	0.11	0.11
**(b) Physical and index properties**											
**Property**	**CL**	**PC**	**FA-F**	**FA-C**	**SL**	**BA**	**RHA**	**GP**	**SF**	**MP**	**MK**
**Specific surface (m^2^/kg)**	-	326	379	290	575	-	1080	382	21,100	519	13,200
**Specific gravity**	2.7	3.15	2.25	2.7	2.88	2.16	2.03	2.54	2.2	2.71	2.6
**Cu (Coefficient of uniformity)**	-	-	8.4	10.7	4.33	4.75	7.1	13.9	-	9.3	-
**Cc (Coefficient of curvature)**	-	-	1.9	2.7	0.47	1.3	1.15	1.55	-	0.76	-

CL = soil (clay); PC = ordinary Portland cement; FA-F = fly ash type F; FA-C = fly ash type C; SL = slag; BA = bottom ash; RHA = rice husk ash; GP = white glass powder; SF = silica fume; MP = marble powder; MK = metakaolin.

**Table 2:** Outline of deep mixing for the control group with Portland cement based grout.

Grout (Binder Only by PC)				Deep Mixing Soil		
w/b	PC (%)	PC (g)	W (g)	Soil (CL) (g)	w (%)	Water (g)
0.85	10	100	85	1000	(LL-5) = 37	370
0.85	15	150	127.5	1000	(LL-5) = 37	370
0.85	20	200	170	1000	(LL-5) = 37	370
1.05	10	100	105	1000	(LL-5) = 37	370
1.05	15	150	157.5	1000	(LL-5) = 37	370
1.05	20	200	210	1000	(LL-5) = 37	370
1.25	10	100	125	1000	(LL-5) = 37	370
1.25	15	150	187.5	1000	(LL-5) = 37	370
1.25	20	200	250	1000	(LL-5) = 37	370
1.25	20	200	250	1000	(LL-10) = 32	320
1.25	20	200	250	1000	(LL-20) = 22	320

w/b = the ratio of water/binder (binder is only Portland cement); PC = ordinary Portland cement (by dry weight of soil); W = weight of water (by dry weight of soil); CL = clayey soil; w = water content.

**Table 3:** Outline of deep mixing for testing group by stabilizer-based geopolymers.

	Geopolymer					Deep Mixing Soil		
Stabilizer	Stabilizer (%)	r = Alkaline Activator/Stabilizer (%)	Stabilizer (g)	Alkaline Activator (g)	NaOH (M)	Soil (CL) (g)	w (%)	Water (g)
	10	0.85	100	85	12 M	1000	(LL-5) = 37	370
	15	0.85	150	127.5	12 M	1000	(LL-5) = 37	370
	20	0.85	200	170	12 M	1000	(LL-5) = 37	370
	25	0.85	250	212.5	12 M	1000	(LL-5) = 37	370
**FA-F**	10	1.05	100	105	12 M	1000	(LL-5) = 37	370
**FA-C**	15	1.05	150	157.5	12 M	1000	(LL-5) = 37	370
**SL**	20	1.05	200	210	12 M	1000	(LL-5) = 37	370
**GP**	25	1.05	250	262.5	12 M	1000	(LL-5) = 37	370
	10	1.25	100	125	12 M	1000	(LL-5) = 37	370
	15	1.25	150	187.5	12 M	1000	(LL-5) = 37	370
	20	1.25	200	250	12 M	1000	(LL-5) = 37	370
	25	1.25	250	312.5	12 M	1000	(LL-5) = 37	370
	25	1.5	250	375	12 M	1000	(LL-5) = 37	370
	20	1.5	200	300	12 M	1000	(LL-5) = 37	370
**25%MP + 75%FA-F**	20	1.05	200	210	12 M	1000	(LL-5) = 37	370
**50%MP + 50%FA-F**	20	1.05	200	210	12 M	1000	(LL-5) = 37	370
**75%MP + 25%FA-F**	20	1.05	200	210	12 M	1000	(LL-5) = 37	370
**50%SL + 50%FA-F**	20	0.85	200	170	12 M	1000	(LL-5) = 37	370
**50%SL + 50%FA-F**	20	1.05	200	210	12 M	1000	(LL-5) = 37	370
**50%SL + 50%FA-F**	20	1.25	200	250	12 M	1000	(LL-5) = 37	370
**15%BA + 85%SL**	20	1.05	200	210	12 M	1000	(LL-5) = 37	370
**30%BA + 70%SL**	20	1.05	200	210	12 M	1000	(LL-5) = 37	370
**45BA% + 55%SL**	20	1.05	200	210	12 M	1000	(LL-5) = 37	370
**60BA% + 40%SL**	20	1.05	200	210	12 M	1000	(LL-5) = 37	370
**75%BA + 25%SL**	20	1.05	200	210	12 M	1000	(LL-5) = 37	370
**15%BA + 85%SL**	20	1.25	200	250	12 M	1000	(LL-5) = 37	370
**30%BA + 70%SL**	20	1.25	200	250	12 M	1000	(LL-5) = 37	370
**45BA% + 55%SL**	20	1.25	200	250	12 M	1000	(LL-5) = 37	370
**60BA% + 40%SL**	20	1.25	200	250	12 M	1000	(LL-5) = 37	370
**75%BA + 25%SL**	20	1.25	200	250	12 M	1000	(LL-5) = 37	370
**100%BA**	20	1.05	200	210	12 M	1000	(LL-5) = 37	370
**100%BA**	20	1.25	200	250	12 M	1000	(LL-5) = 37	370
**10%RHA + 90%SL**	20	1.05	200	210	12 M	1000	(LL-5) = 37	370
**20%RHA + 80%SL**	20	1.05	200	210	12 M	1000	(LL-5) = 37	370
**30%RHA + 70%SL**	20	1.05	200	210	12 M	1000	(LL-5) = 37	370
**10%RHA + 90%SL**	20	1.25	200	250	12 M	1000	(LL-5) = 37	370
**20%RHA + 80%SL**	20	1.25	200	250	12 M	1000	(LL-5) = 37	370
**30%RHA + 70%SL**	20	1.25	200	250	12 M	1000	(LL-5) = 37	370
**40%RHA + 60%SL**	20	1.25	200	250	12 M	1000	(LL-5) = 37	370
**50%GP + 50%SL**	20	1.05	200	210	12 M	1000	(LL-5) = 37	370
**12.5%SF + 87.5%SL**	20	1.05	200	210	12 M	1000	(LL-5) = 37	370
**12.5%SF + 87.5%SL**	20	1.25	200	250	12 M	1000	(LL-5) = 37	370
**25%SF + 75%SL**	20	1.25	200	250	12 M	1000	(LL-5) = 37	370
**37.5%SF + 62.5%SL**	20	1.25	200	250	12 M	1000	(LL-5) = 37	370
**50%SF + 50%SL**	20	1.25	200	250	12 M	1000	(LL-5) = 37	370
**MK**	10	1.25	100	125	12 M	1000	(LL-5) = 37	370
**MK**	15	1.25	150	187.5	12 M	1000	(LL-5) = 37	370
**MK**	20	1.25	200	250	12 M	1000	(LL-5) = 37	370
**MK**	25	1.25	250	312.5	12 M	1000	(LL-5) = 37	370
**MK**	30	1.25	300	375	12 M	1000	(LL-5) = 37	370
**12.5%MK + 87.5%SL**	20	1.25	200	250	12 M	1000	(LL-5) = 37	370
**25%MK + 75%SL**	20	1.25	200	250	12 M	1000	(LL-5) = 37	370
**37.5%MK + 62.5%SL**	20	1.25	200	250	12 M	1000	(LL-5) = 37	370
**50%MK + 50%SL**	20	1.25	200	250	12 M	1000	(LL-5) = 37	370
**GP**	15	1.05	150	157.5	14 M	1000	(LL-5) = 37	370
**FA-F**	15	1.05	15	157.5	14 M	1000	(LL-5) = 37	370
**FA-C**	20	1.05	200	210	14 M	1000	(LL-5) = 37	370
**SL**	20	1.25	200	250	14 M	1000	(LL-5) = 37	370
**GP**	15	1.05	150	157.5	10 M	1000	(LL-5) = 37	370
**FA-F**	15	1.05	15	157.5	10 M	1000	(LL-5) = 37	370
**FA-C**	20	1.05	200	210	10 M	1000	(LL-5) = 37	370
**SL**	20	1.25	200	250	10 M	1000	(LL-5) = 37	370
**GP**	15	1.05	150	157.5	8 M	1000	(LL-5) = 37	370
**FA-F**	15	1.05	15	157.5	8 M	1000	(LL-5) = 37	370
**FA-C**	20	1.05	200	210	8 M	1000	(LL-5) = 37	370
**SL**	20	1.25	200	250	8 M	1000	(LL-5) = 37	370
**GP**	15	1.05	150	157.5	16 M	1000	(LL-5) = 37	370
**FA-F**	15	1.05	15	157.5	16 M	1000	(LL-5) = 37	370
**FA-C**	20	1.05	200	210	16 M	1000	(LL-5) = 37	370
**SL**	20	1.25	200	250	16 M	1000	(LL-5) = 37	370
**GP**	15	1.05	150	157.5	12 M	1000	(LL-10) = 32	320
**GP**	15	1.05	150	157.5	12 M	1000	(LL-20) = 22	220
**FA-F**	15	1.05	150	157.5	12 M	1000	(LL-10) = 32	320
**FA-F**	15	1.05	150	157.5	12 M	1000	(LL-20) = 22	220
**FA-C**	20	1.05	200	210	12 M	1000	(LL-10) = 32	320
**FA-C**	20	1.05	200	210	12 M	1000	(LL-20) = 22	220
**SL**	20	1.25	200	250	12 M	1000	(LL-10) = 32	320
**SL**	20	1.25	200	250	12 M	1000	(LL-20) = 22	220

Geopolymer = stabilizer + alkaline activator; Alkaline activator = NaOH + Na_2_SiO_3_; Stabilizer (%) = by dry weight of soil; Alkaline Activator/stabilizer (%) = Alkaline Activator/Stabilizer by dry weight; CL = clay; w = water content.

## References

[1] Bowles J.E. (1996). Foundation Analysis and Design.

[2] Coduto D.P. (1999). Geotechnical Engineering: Principles and Practices.

[3] Hasheminezhad A., Bahadori H. (2019). Seismic response of shallow foundations over liquefiable soils improved by deep soil mixing columns. Comput. Geotech..

[4] Kitazume M., Terashi M. (2013). The Deep Mixing Method.

[5] Porbaha A. (1998). State of the art in deep mixing technology: part I. Basic concepts and overview. Proceedings of the Institution of Civil Engineers-Ground Improvement.

[6] Frikha W., Zargayouna H., Boussetta S., Bouassida M. (2017). Experimental study of Tunis soft soil improved by deep mixing column. Geotech. Geol. Eng..

[7] Khedari J., Watsanasathaporn P., Hirunlabh J. (2005). Development of fibre-based soil–cement block with low thermal conductivity. Cem. Concr. Compos..

[8] Cristelo N., Glendinning S., Teixeira Pinto A. (2011). Deep soft soil improvement by alkaline activation. Proceedings of the Institution of Civil Engineers-Ground Improvement.

[9] Cristelo N., Glendinning S., Fernandes L., Pinto A.T. (2012). Effect of calcium content on soil stabilisation with alkaline activation. Constr. Build. Mater..

[10] Arulrajah A., Yaghoubi M., Disfani M.M., Horpibulsuk S., Bo M.W., Leong M. (2018). Evaluation of fly ash-and slag-based geopolymers for the improvement of a soft marine clay by deep soil mixing. Soils Found..

[11] Zhang M., Guo H., El-Korchi T., Zhang G., Tao M. (2013). Experimental feasibility study of geopolymer as the next-generation soil stabilizer. Constr. Build. Mater..

[12] Davidovits J. (2015). Geopolymer Chemistry and Applications.

[13] El Idrissi A.C., Roziere E., Loukili A., Darson S. (2018). Design of geopolymer grouts: the effects of water content and mineral precursor. Eur. J. Environ. Civ. Eng..

[14] Kuo W.T., Liu M.Y., Juang C.U. (2019). Bonding behavior of repair material using fly-ash/ground granulated blast furnace slag-based geopolymer. Materials.

[15] Xie J., Zhao J., Wang J., Wang C., Huang P., Fang C. (2019). Sulfate resistance of recycled aggregate concrete with GGBS and fly ash-based geopolymer. Materials.

[16] Wang H., Li H., Yan F. (2005). Synthesis and mechanical properties of metakaolinite-based geopolymer. Colloids Surf. A.

[17] Pacheco-Torgal F., Moura D., Ding Y., Jalali S. (2011). Composition, strength and workability of alkali-activated metakaolin based mortars. Constr. Build. Mater..

[18] Yunsheng Z., Wei S., Zongjin L. (2010). Composition design and microstructural characterization of calcined kaolin-based geopolymer cement. Appl. Clay Sci..

[19] Phetchuay C., Horpibulsuk S., Suksiripattanapong C., Chinkulkijniwat A., Arulrajah A., Disfani M.M. (2014). Calcium carbide residue: Alkaline activator for clay–fly ash geopolymer. Constr. Build. Mater..

[20] Phetchuay C., Horpibulsuk S., Arulrajah A., Suksiripattanapong C., Udomchai A. (2016). Strength development in soft marine clay stabilized by fly ash and calcium carbide residue based geopolymer. Appl. Clay Sci..

[21] Yi Y., Li C., Liu S. (2014). Alkali-activated ground-granulated blast furnace slag for stabilization of marine soft clay. J. Mater. Civ. Eng..

[22] Rios S., Cristelo N., Viana da Fonseca A., Ferreira C. (2015). Structural performance of alkali-activated soil ash versus soil cement. J. Mater. Civ. Eng..

[23] Singhi B., Laskar A.I., Ahmed M.A. (2016). Investigation on soil–geopolymer with slag, fly ash and their blending. Arab. J. Sci. Eng..

[24] Du Y.J., Yu B.W., Liu K., Jiang N.J., Liu M.D. (2016). Physical, hydraulic, and mechanical properties of clayey soil stabilized by lightweight alkali-activated slag geopolymer. J. Mater. Civ. Eng..

[25] Singhi B., Laskar A.I., Ahmed M.A. (2017). Mechanical behavior and sulfate resistance of alkali activated stabilized clayey soil. Geotech. Geol. Eng..

[26] Samson G., Cyr M., Gao X.X. (2017). Formulation and characterization of blended alkali-activated materials based on flash-calcined metakaolin, fly ash and GGBS. Constr. Build. Mater..

[27] Bilondi M.P., Toufigh M.M., Toufigh V. (2018). Experimental investigation of using a recycled glass powder-based geopolymer to improve the mechanical behavior of clay soils. Constr. Build. Mater..

[28] Bilondi M.P., Toufigh M.M., Toufigh V. (2018). Using calcium carbide residue as an alkaline activator for glass powder-clay geopolymer. Constr. Build. Mater..

[29] Aboulayt A., Jaafri R., Samouh H., El Idrissi A.C., Roziere E., Moussa R., Loukili A. (2018). Stability of a new geopolymer grout: rheological and mechanical performances of metakaolin-fly ash binary mixtures. Constr. Build. Mater..

[30] Ghadir P., Ranjbar N. (2018). Clayey soil stabilization using geopolymer and Portland cement. Constr. Build. Mater..

[31] Leong H.Y., Ong D.E.L., Sanjayan J.G., Nazari A. (2018). Strength development of soil–fly ash geopolymer: assessment of soil, fly ash, alkali activators, and water. J. Mater. Civ. Eng..

[32] Güllü H., Cevik A., Al-Ezzi K.M., Gülsan M.E. (2019). On the rheology of using geopolymer for grouting: A comparative study with cement-based grout included fly ash and cold bonded fly ash. Constr. Build. Mater..

[33] ASTM D2487-11 (2011). Standard Practice for Classification of Soils for Engineering Purposes.

[34] Bhadriraju V., Puppala A.J., Madhyannapu R.S., Williammee R. (2007). Laboratory procedure to obtain well-mixed soil binder samples of medium stiff to stiff expansive clayey soil for deep soil mixing simulation. Geotech. Test. J..

[35] Pakbaz M.S., Farzi M. (2015). Comparison of the effect of mixing methods (dry vs. wet) on mechanical and hydraulic properties of treated soil with cement or lime. Appl. Clay Sci..

[36] ASTM C150/C150M-17 (2017). Standard Specification for Portland cement.

[37] ASTM C618-15 (2015). Standard Specification for Coal Fly Ash and Raw or Calcined Natural Pozzolan for Use in Concrete.

[38] Celik F., Canakci H. (2018). Examination of the mechanical properties and failure pattern of soilcrete mixtures modified with rice husk ash. Eur. J. Environ. Civ. Eng..

[39] Wen N., Zhao Y., Yu Z., Liu M. (2019). A sludge and modified rice husk ash-based geopolymer: synthesis and characterization analysis. J. Clean. Prod..

[40] ASTM C1240-15 (2015). Standard Specification for Silica Fume Used in Cementitious Mixtures.

[41] ACI, 234R-96, 1996 (1996). Guide for the use of Silica Fume in Concrete.

[42] Cevik A., Alzeebaree R., Humur G., Nis A., Gulsan M.E. (2018). Effect of nano-silica on the chemical durability and mechanical performance of fly ash based geopolymer concrete. Ceram. Int..

[43] Mohd Ali A.Z., Sanjayan J., Guerrieri M. (2017). Performance of geopolymer high strength concrete wall panels and cylinders when exposed to a hydrocarbon fire. Constr. Build. Mater..

[44] Güllü H., Canakci H., Al Zangana I.F. (2017). Use of cement based grout with glass powder for deep mixing. Constr. Build. Mater..

[45] JGS 0821–2000 (2000). Practice for Marking and Curing Stabilized Soil Specimens without Compaction.

[46] ASTM D2166 (2016). Standard Test Method for Unconfined Compressive Strength of Cohesive Soil.

[47] ASTM D5102-09 (2009). Standard Test Methods for Unconfined Compressive Strength of Compacted Soil-Lime Mixtures.

[48] ASTM C597-16 (2016). Standard Test Method for Pulse Velocity through Concrete.

[49] Terashi M., Kitazume M. (2011). QA/QC for deep-mixed ground: current practice and future research needs. Proceedings of the Institution of Civil Engineers-Ground Improvement.

[50] Abbey S.J., Ngambi S., Ngekpe B.E. (2015). Understanding the performance of deep mixed column improved soils-a review. Int. J. Civ. Eng. Technol. (Ijciet).

[51] Anon O.H. (1979). Classification of rocks and soils for engineering geological mapping, Part 1—rock and soil materials. Report of the Commission of Engineering Geological Mapping.

[52] Patel Y.J., Shah N. (2018). Enhancement of the properties of ground granulated blast furnace slag based self compacting geopolymer concrete by incorporating rice husk ash. Constr. Build. Mater..

[53] Temuujin J.V., Van Riessen A., Williams R. (2009). Influence of calcium compounds on the mechanical properties of fly ash geopolymer pastes. J. Hazard. Mater..

[54] Misra A., Biswas D., Upadhyaya S. (2005). Physico-mechanical behavior of self-cementing class C fly ash-clay mixtures. Fuel.

[55] Nath P., Sarker P.K. (2014). Effect of GGBFS on setting, workability and early strength properties of fly ash geopolymer concrete cured in ambient condition. Constr. Build. Mater..

[56] Vafaei M., Allahverdi A. (2017). High strength geopolymer binder based on waste-glass powder. Adv. Powder Technol..

[57] Jain N. (2012). Effect of nonpozzolanic and pozzolanic mineral admixtures on the hydration behavior of ordinary Portland cement. Constr. Build. Mater..

[58] Xu H., Gong W., Syltebo L., Izzo K., Lutze W., Pegg I.L. (2014). Effect of blast furnace slag grades on fly ash based geopolymer waste forms. Fuel.

[59] Rafieizonooz M., Mirza J., Salim M.R., Hussin M.W., Khankhaje E. (2016). Investigation of coal bottom ash and fly ash in concrete as replacement for sand and cement. Constr. Build. Mater..

[60] Hwang C.L., Huynh T.P. (2015). Effect of alkali-activator and rice husk ash content on strength development of fly ash and residual rice husk ash-based geopolymers. Constr. Build. Mater..

[61] Duan P., Yan C., Zhou W. (2017). Compressive strength and microstructure of fly ash based geopolymer blended with silica fume under thermal cycle. Cem. Concr. Compos..

[62] Chindaprasirt P., Paisitsrisawat P., Rattanasak U. (2014). Strength and resistance to sulfate and sulfuric acid of ground fluidized bed combustion fly ash-silica fume alkali-activated composite. Adv. Powder Technol..

[63] Hasnaoui A., Ghorbel E., Wardeh G. (2019). Optimization approach of granulated blast furnace slag and metakaolin based geopolymer mortars. Constr. Build. Mater..

[64] Kawasaki T., Niina A., Saitoh S., Suzuki Y., Honjo Y. (1981). Deep mixing method using cement hardening agent. Proceedings of the 10th International Conference on Soil Mechanics and Foundation Engineering.

[65] Hassan M. (2009). Engineering Characteristics of Cement Stabilized Soft Finnish Clay-a Laboratory Study Licentiate Thesis.

[66] Kramer S.L. (1996). Geotechnical Earthquake Engineering.

[67] Mandal T., Tinjum J.M., Edil T.B. (2016). Non-destructive testing of cementitiously stabilized materials using ultrasonic pulse velocity test. Transp. Geotech..

[68] Khale D., Chaudhary R. (2007). Mechanism of geopolymerization and factors influencing its development: A review. J. Mater. Sci..

[69] Hardjito D. (2005). Studies of Fly Ash-based Geopolymer Concrete. Ph.D. Thesis.

[70] Chindaprasirt P., Jaturapitakkul C., Chalee W., Rattanasak U. (2009). Comparative study on the characteristics of fly ash and bottom ash geopolymers. Waste Manag..

[71] Somna K., Jaturapitakkul C., Kajitvichyanukul P., Chindaprasirt P. (2011). NaOH-activated ground fly ash geopolymer cured at ambient temperature. Fuel.

[72] Lorenzo G.A., Bergado D.T. (2006). Fundamental characteristics of cement-admixed clay in deep mixing. J. Mater. Civ. Eng..

[73] Basu A., Mishra D.A., Roychowdhury K. (2013). Rock failure modes under uniaxial compression, Brazilian, and point load tests. Bull. Eng. Geol. Environ..

[74] Illampas R., Ioannou I., Charmpis D.C. (2014). Adobe bricks under compression: experimental investigation and derivation of stress–strain equation. Constr. Build. Mater..

[75] Fernández-Jiménez A., Palomo A. (2005). Composition and microstructure of alkali activated fly ash binder: Effect of the activator. Cem. Concr. Res..

[76] Xu W., Lo T.Y., Memon S.A. (2012). Microstructure and reactivity of rich husk ash. Constr. Build. Mater..

[77] He J., Jie Y., Zhang J., Yu Y., Zhang G. (2013). Synthesis and characterization of red mud and rice husk ash-based geopolymer composites. Cem. Concr. Compos..

[78] Liew Y.M., Kamarudin H., Al Bakri A.M., Luqman M., Nizar I.K., Ruzaidi C.M., Heah C.Y. (2012). Processing and characterization of calcined kaolin cement powder. Constr. Build. Mater..

